# Switching from Oral Cholinesterase Inhibitors to a Transdermal Donepezil Patch Attenuated Gastrointestinal Symptoms and Allowed Treatment Continuation in Three Patients with Alzheimer’s Disease in Clinical Settings

**DOI:** 10.3390/brainsci16010098

**Published:** 2026-01-17

**Authors:** Yumiko Motoi, Nobuo Sanjo

**Affiliations:** 1 The Medical Center for Dementia, Juntendo Hospital, Tokyo 113-0033, Japan; 2 Department of Therapeutics for Dementia, Juntendo University, Tokyo 113-8421, Japan; 3 Department of Neurology, Juntendo University, Tokyo 113-8421, Japan; 4 Department of Neurology and Neurological Sciences, Institute of Science Tokyo, Tokyo 152-8550, Japan

**Keywords:** cholinesterase inhibitors, Alzheimer’s disease, donepezil, transdermal patch

## Abstract

**Background**: Cholinesterase inhibitors (ChEIs) are commonly prescribed for the treatment of Alzheimer’s disease (AD) and achieve long-term benefits for cognition and survival in real-world settings. However, the discontinuation rate is high due to their side effects, with gastrointestinal (GI) symptoms hampering long-term prescriptions. The risk of side effects associated with rivastigmine was previously shown to be lower with transdermal delivery than with oral capsules; however, this has yet to be examined in detail for donepezil, the most widely used ChEI. The daily application of a donepezil transdermal patch was officially approved in Japan in 2023. The incidence of side effects was lower with the donepezil transdermal patch than with oral donepezil in healthy volunteers, but has not yet been assessed in clinical settings. **Results**: We herein report three AD patients in two different memory clinics who developed GI symptoms with oral ChEIs that were attenuated by switching to the donepezil transdermal patch. **Conclusions**: The donepezil transdermal patch may improve tolerability and adherence in patients who develop gastrointestinal adverse effects with oral donepezil.

## 1. Introduction

The number of people living with dementia was estimated to be 55 million in 2019 and is expected to increase to 139 million by 2050 [1]. Alzheimer’s disease (AD) represents the most common cause of dementia worldwide and is characterized by amyloid-β plaques and tau neurofibrillary tangles [2,3]. Although two new anti-amyloid-β antibodies, lecanemab and donanemab, have recently been approved and represent a critical milestone, these drugs are only available for mild cognitive impairment (MCI) and mild dementia due to AD [4,5]. Therefore, acetyl-cholinesterase inhibitors (ChEIs) still represent the main pharmacological treatment for AD, particularly the moderate and severe stages. A recent review of post-marketing open/non-randomized/retrospective studies described the long-term effects (4-year projection) in AD patients treated with ChEIs, with the mean Mini-Mental State Examination (MMSE) loss ranging from 0.2 to 1.37 points/years, in contrast to 1.07–3.4 points/years in non-treated patients [6]. Furthermore, a reduction in the relative risk of mortality of between 27 and 42% was observed over a period of 2 to 8 years [6]. Therefore, long-term treatment with ChEIs represents a beneficial strategy in real-world settings.

Three ChEIs are currently available, donepezil, rivastigmine, and galantamine, but have high discontinuation rates [7]. Previous studies reported fewer side effects with donepezil, the most commonly used ChEI, than with rivastigmine and galantamine [8,9]. Nevertheless, a 1-year observational study on 398 participants from 38 institutions in seven Asian countries showed that the discontinuation rate of donepezil was high at 20.9% with a mean treatment duration of 103.67 days [10]. In a 48-week observational study on 117 Japanese AD patients, the discontinuation rate of donepezil was approximately 36% and was not reduced by a psychoeducational intervention [11]. A nationwide study on community-dwelling individuals with clinically verified AD (n = 6858) in Spain showed that around 20% of ChEI users discontinued treatment within the first year [12]. In a cohort of 1999 new users of donepezil aged 65 years or older in New Zealand, the discontinuation rate was 49% in a 12-month period [13]. A population-based cohort study using British Columbia claims data assessed new ChEI users aged 40 years and older and found that 12,845 (52%) discontinued therapy or died within 1 year [14]. Based on these findings, 20–50% of patients who started ChEIs discontinued treatment after less than 1 year.

The most common reason for discontinuation was the occurrence of an adverse event [10]. AChE inhibitors are commonly associated with classic cholinergic side effects, such as nausea, diarrhea, vomiting, dizziness, and bradycardia [15]. The gastrointestinal (GI) peripheral side effects of ChEIs may be attributed to the exposure of GI organs to acetylcholine. GI symptoms are the most common side effect [16], and include nausea, vomiting, a loss of appetite, and an increased frequency of bowel movements [8]. The incidence of nausea in patients receiving donepezil was previously shown to range between 8.3 and 24.1%. Comparisons of donepezil (10 mg/day) and rivastigmine (3–12 mg/day) revealed that nausea was a less frequent side effect with the former [8]. Furthermore, after 6 months of treatment, vomiting was reported as a side effect in 6.9–15.7% of patients receiving donepezil, 9.8–20.4% of those receiving galantamine, and 21.5–38.5% of those receiving rivastigmine [8]. Since the incidence of nausea and vomiting in this study was the highest with rivastigmine capsules, transdermal patches were developed [8]. A previous study demonstrated that the incidence of side effects associated with rivastigmine was two-thirds lower with its transdermal delivery via a 10-cm^2^ patch than with oral capsules [17]; however, the effects of transdermal donepezil patches have not yet been examined in detail.

The daily application of Allydone Patches, a tape formulation for the transdermal delivery of donepezil, was approved for the treatment of AD by the Ministry of Health Labour and Welfare of Japan in 2023 [18]. Non-inferiority in the suppression of cognitive decline was shown for the 27.5 mg transdermal donepezil patch when compared with 5 mg donepezil hydrochloride tablets in Japanese patients with mild-to-moderate AD. A phase 1, open-label, single-center, multiple-dose study showed that the pharmacokinetics of the repeated application of the 27.5 mg transdermal donepezil patch was similar to that of 5 mg donepezil oral tablets as assessed by AUC_0–24h_ [19]. The donepezil transdermal patches that are applied weekly were also approved in the United States by the Food and Drug Administration in 2022. The incidence of cholinergic GI side effects is expected to be lower with these patches than with oral capsules, similar to rivastigmine, due to more gradual increases in donepezil levels in the blood [20,21]. While this has already been demonstrated in healthy volunteers [22], it has not yet been examined in clinical settings. Therefore, we herein report three AD patients in two different memory clinics who developed GI symptoms with oral donepezil that were resolved by switching to donepezil transdermal patches.

## 2. Case Presentation (Table 1)

**Figure 1:**
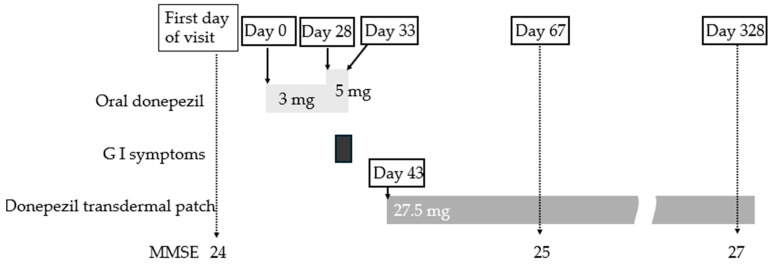
The timeline of oral and transdermal donepezil administration and the development of gastrointestinal symptoms in Case 1. Nausea was observed from the day after the dose of donepezil was increased to 5 mg until the day after its discontinuation (black square). The day of starting 3 mg donepezil was defined as Day 0. First day of visit is 24 days before the day of starting 3 mg of donepezil. GI symptoms indicate gastrointestinal symptoms.

**Case 1**: An 83-year-old woman visited memory clinic A with forgetfulness. She had no comorbidities other than a history of cataract surgery and was not taking any regular medications. Her husband noticed that she was unable to remember her bank account PIN and had to be contacted by the bank. She also often searched for things at home. Her Mini-Mental State Examination (MMSE) and Montreal Cognitive Assessment (MoCA) test scores were 24 and 18, respectively. Cranial MRI showed no abnormalities; however, regional cerebral blood flow SPECT with the 99mTc-ethyl cysteinate dimer (PDRadiopharma Inc., Japan, Tokyo) revealed low perfusion of the posterior cingulate gyrus and lateral parietal lobe, consistent with the abnormal pattern of AD. Therefore, the patient was diagnosed with AD and initially prescribed 3 mg oral donepezil for 14 days followed by a dose escalation to 5 mg (Figure 1). Nausea developed on the second day after the dose of donepezil was increased to 5 mg. There was no vomiting, and the patient was able to maintain an oral intake of food and fluids; however, dietary intake decreased to approximately half of the pre-titration level. Therefore, on day 6, donepezil was discontinued according to the physician’s instructions. There was no change in body weight and no signs of dehydration. Bowel movements were normal, and no additional medications were administered and no additional gastrointestinal examinations were performed. Nausea began to improve the day after the discontinuation of oral donepezil, and dietary intake returned to the pre-medication level two days later. Ten days after the discontinuation of donepezil, a 27.5 mg donepezil transdermal patch was initiated. The physician instructed the patient to apply the transdermal patch to the back or upper arm, rotating the application site daily, and advised that the patient’s husband apply the patch if possible. Her husband faithfully followed these instructions and applied the patch to a different site each day. No skin reactions, such as erythema or pruritus, were observed. The patient continued the transdermal therapy and repeat MMSE assessments performed approximately 3 months and 1 year later showed no decline.

**Table 1:** Clinical characteristics of the three cases.

Characteristic	Case 1	Case 2	Case 3
Age	83	86	75
Sex	Female	Female	Female
MMSE	24	22	28
MOCA test	18	14	Not examined
Prior prescription	5 mg oral donepezil	5 mg oral donepezil 9 mg rivastigmine patch	5 mg oral donepezil
Time to switch	1 month	1 year	1 year
Gastrointestinal symptom	Nausea	Diarrhea	Epigastric discomfort

In Japan, 5 mg donepezil is indicated for mild and moderate AD and 10 mg donepezil for severe AD.

**Case 2**: An 86-year-old woman visited memory clinic B with forgetfulness. Her physician diagnosed MCI and she was continuously monitored. One year later, cognitive function worsened, and she started to forget her bank account PIN and address. She visited the memory clinic with her children, who also noticed that she became easily irritated with them. MMSE and MoCA test scores were 22 and 14, respectively. Brain MRI revealed atrophy of the bilateral medial temporal lobes. Wechsler memory scale tests showed severe impairments in visual and verbal memory functions. Based on these findings, she was diagnosed with dementia due to AD. As treatment, 5 mg of donepezil was initiated after a 3 mg induction period for 2 weeks. No other medication was prescribed. The patient developed diarrhea once or twice a day and had no abdominal pain or nausea/vomiting. Although no signs of dehydration were observed, oral donepezil was discontinued at the discretion of the patient and her family, and the diarrhea resolved 2–3 days after discontinuation. When the patient visited the outpatient clinic several days later, her appetite had recovered; therefore, no medications or examinations for gastrointestinal symptoms were administered. The prescription was changed to a 9 mg rivastigmine patch, which only slightly attenuated diarrhea. One year after changing to rivastigmine, her MoCA test score decreased to 11 and, thus, a 27.5 mg donepezil transdermal patch was prescribed. The physician instructed the patient and her family to rotate the patch application site between the back and upper arm. No gastrointestinal or skin-related adverse events were observed, and cognitive function remained stable. Six months after changing to the donepezil patch, her MMSE score was 22. Since the patient lived alone, she applied the patch herself with her daughter sometimes applying it to her arm. The patient reported that her duration of sleep during the day decreased and her activity level slightly increased.

**Figure 2:**
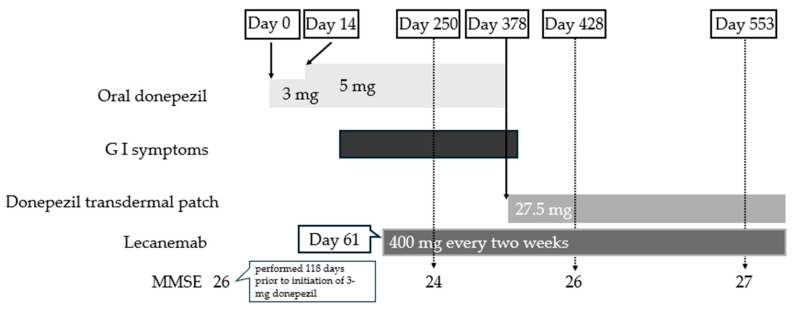
The timeline of oral and transdermal donepezil administration, lecanemab treatment, and the development of gastrointestinal symptoms in Case 3. Gastric discomfort persisted from several days after initiation of donepezil at 5 mg until several days after switching to the donepezil transdermal patch (black square). The day of starting 3 mg donepezil was defined as Day 0. GI symptoms indicate gastrointestinal symptoms.

**Case 3**: A 75-year-old woman visited memory clinic A with forgetfulness. Her past medical history included mastopathy, cholecystectomy, and herpes zoster, and she was currently taking risedronate for osteoporosis. She scored 28 for MMSE and MRI and regional cerebral blood SPECT were unremarkable. Her physician made a diagnosis of MCI. One year after the initial visit, the MMSE score had slightly declined to 26 points, and the patient increasingly repeated the same questions to her husband. After lecanemab was approved, amyloid PET imaging was performed and yielded a positive result (the Centiloid scale was 72); consequently, the diagnosis recorded in the medical chart was revised to AD. Donepezil was initiated at 3 mg and increased to 5 mg after 14 days (Figure 2). Several days after the dose escalation, the patient developed gastric discomfort; however, this did not progress to nausea or a marked loss of appetite, and she did not specifically report these symptoms to her physician. No medications or diagnostic examinations were undertaken for these symptoms.

Two months after the initiation of donepezil, lecanemab therapy was started. On the first day of lecanemab administration, the patient complained of headache, which resolved after the oral administration of acetaminophen (200 mg). Gastric discomfort persisted thereafter, and after nearly 1 year of lecanemab treatment, she continued to receive biweekly intravenous lecanemab infusions and experienced no other serious adverse events, including amyloid-related imaging abnormalities (ARIA). Feeling reassured, she then informed her physician of the epigastric discomfort. Considering the possibility of donepezil-related adverse effects, the physician switched the treatment to a 27.5 mg donepezil transdermal patch without a washout period. When a nurse explained to both the patient and her husband, using the instruction leaflet, that the patch should be changed daily and applied to different sites on the back or upper arm, the husband began to apply the patch daily in accordance with the instructions, rotating the application site.

Several days after switching to the transdermal patch, the patient no longer complained of gastric discomfort and began traveling more frequently. Because mild skin reactions, including erythema and pruritus, were observed, flumetasone lotion was applied. The clinical time course suggested that lecanemab was unlikely to be associated with the gastrointestinal symptoms. The MMSE score remained stable at 27 points at 553 days after initiation of donepezil and 492 days after initiation of lecanemab.

## 3. Discussion

At two different memory clinics, three AD patients were initially prescribed oral ChEIs and developed GI symptoms. After switching to donepezil transdermal patches, these symptoms resolved in all three patients. These cases were selected based on clinical experience rather than as consecutive cases meeting predefined criteria. The descriptions of these three case reports were prepared in accordance with the CARE case report guidelines [23]. Gastrointestinal symptoms differed among the three cases in terms of symptom type, onset pattern, and severity. In Case 1, nausea and decreased appetite developed 2–3 days after the dose was increased to 5 mg; the duration of oral donepezil treatment was short (less than one month), and the patient visited the clinic without waiting for the next scheduled appointment. In Case 2, the medication was also self-discontinued without awaiting the physician’s decision. In contrast, in Case 3, because the gastrointestinal symptoms were limited to mild gastric discomfort, oral donepezil at 5 mg was continued for one year. After switching to the transdermal formulation, cutaneous adverse reactions developed; however, continued use of the patch was possible with topical steroid application by the caregiver. Overall, these three cases may be considered examples in which tolerability and adherence improved as a result of the alleviation of gastrointestinal symptoms.

Two mechanisms for the attenuation of GI side effects with the transdermal delivery of ChEIs have been proposed. On the one hand, the GI side effects of ChEIs for AD may be attributed to the direct exposure of GI organs to acetylcholine. Therefore, bypassing the GI tract may prevent organs from being exposed to these drugs [16]. On the other hand, transdermal patches have the advantage of increasing blood levels of the active drug more gradually than the oral formulation, thereby providing more stable blood levels. The phase 1 open-label study conducted in healthy Japanese subjects showed that on the first day, median T max for a transdermal patch with a donepezil dosage of 27.5 mg was 24.08 h, while that for 5 mg oral donepezil was only 4.00 h [18,19]. On day 1, the maximum plasma concentration (Cmax) was 3.018 ng/mL with the transdermal formulation and 2.843 ng/mL with the oral formulation although this study showed similar pharmacokinetics of donepezil transdermal patches and oral donepezil.

Four prospective studies and one retrospective analysis have described the efficacy and safety of switching from oral ChEIs to transdermal rivastigmine patches (Table 2). MMSE scores did not significantly change after the switch in the 4 prospective studies [24,25,26,27], however in the group with a lack/loss of response to oral ChEIs, the decline of MMSE score improved from −3.4 ± 2.5 points in the 6 months before switching to −0.5 ± 3.2 after [26]. Moreover, the incidence of GI symptoms after the switch to the transdermal rivastigmine patch ranged between 0.8 and 13% [24,25,26,27], which was lower than that with oral donepezil (8.3–24.1%) [8]. However, a retrospective cohort study examined 772 patients who were new donepezil users and subsequently switched to the rivastigmine patch, and showed no significant differences in GI complications [28]. The effects of switching from 5 mg oral donepezil to an IPI-301 donepezil transdermal patch (applied twice weekly) were also investigated in a prospective, randomized, and double-blind large-scale study; however, GI side effects were not described [29]. Since these studies did not clearly show that switching from oral ChEIs to transdermal ChEIs decreased GI side effects, the findings of the present cases will be very helpful for decision-making by physicians in clinical settings.

Long-term ChEI treatment has been associated with longer survival [6]; however, the prevalence of their use and compliance are not consistent worldwide. In Hungary, 61,369 patients with dementia selected from the NEUROHUN database, which included all in- and outpatient medical records, were analyzed between 2013 and 2016 [30]. The findings obtained showed that median survival was 2.50 years in 51,875 ChEI prescription non-fillers, while in the case of 8803 ChEI prescription fillers, although it was not possible to assess the median during the analyzed period, it was longer than 4 years. In Japan, a retrospective analysis of the Tajiri project identified 100 patients diagnosed with AD from 390 subjects with medical records and death certificates between 1999 and 2012, and revealed that lifetime expectancies after the onset of AD were 7.9 years in 52 patients treated with donepezil and 5.3 years in 48 patients not treated with donepezil [31]. A cohort study on 7073 subjects diagnosed with AD from the Swedish Dementia Registry showed that the use of ChEIs was associated with a lower risk of death (HR: 0.64, 95% CI: 0.54–0.76) [32]. Chronic vagal nerve stimulation by donepezil may improve long-term survival through cardioprotection [33]. Treatment that promotes cholinergic function in patients with AD may also have more durable beneficial biological effects on the brain than a temporary augmentation of cognitive function [34]. The long-term maintenance of patients prescribed CHEIs without side effects represents an important goal.

This study has several limitations inherent to case reports. Because of the small sample size, the findings cannot be generalized. Larger-scale randomized studies switching from oral donepezil to donepezil transdermal patch and prospective cohort studies are required to confirm the tolerability and clinical benefits observed in this report.

## 4. Conclusions

We herein described three AD patients with attenuated GI side effects after switching from oral to transdermal donepezil. In patients with AD who develop gastrointestinal symptoms during oral ChEI therapy, switching to transdermal ChEIs may improve tolerability and adherence, allowing the long-term use of donepezil.

## Figures and Tables

**Table 2:** Summary of studies switching from oral CHEIs to transdermal rivastigmine patches.

Published Authors and Date	Type of the Study	Sample Size (n)	Baseline MMSE Score	Previous Oral ChEIs	Dose of Rivastigmine Patch After the Switch	Follow-Up	MMSE Change from Baseline	GI-Related Side Effects of Rivastigmine Patches
Ueda K et al., 2019 [25]	Prospective, open- label, 1-step titration	118	17.3	Don, Gal	9 mg/5 cm^2^–18 mg/10 cm^2^	24 Ws	−0.36 (*p* = 0.18)	7.6%
Cagnin A et al., 2015 [26]	Prospective, open- label, single arm	174	17.4	Don, Riv	4.6 mg/5 cm^2^	6 Ms	−0.5 ± 3.2	13%
Han et al., 2011 [24]	Prospective, open- label, single arm,	164	17.2	Don, Gal, Riv	5–10 cm^2^	24 Ws	0.4 (*p* > 0.05)	1.2%
Sadowsky et al., 2009 [27]	Prospective, open- label	261	18.3	Don	4.6 mg/5 cm^2^	5 Ws	n.d.	0.8–3.8%
Tian H 2013 [28]	Retrospective cohort	772	n.d.	Don	n.d.	-	n.d.	No difference

n.d.: not described, Don: Donepezil, Gal: Galantamine, Riv: Rivastigmine, Ws: weeks, Ms: months.

## Data Availability

The original contributions presented in this study are included in the article. Further inquiries can be directed to the corresponding author.
